# Quasi-Static Modelling of a Full-Channel Effective Magnetorheological Damper with Trapezoidal Magnetic Rings

**DOI:** 10.3390/ma16206820

**Published:** 2023-10-23

**Authors:** Huan Wu, Yiming Hu, Yinong Li, Sanbao Gu, Ziyang Yue, Xiaoxue Yang, Ling Zheng

**Affiliations:** 1 State Key Laboratory of Mechanical Transmission, Chongqing University, Chongqing 400030, China; 201934131007@cqu.edu.cn (H.W.); 202207131505@stu.cqu.edu.cn (Z.Y.);; 2 School of Mechatronics and Vehicle Engineering, East China Jiaotong University, Nanchang 330013, China; huyiming139@hotmail.com

**Keywords:** magnetorheological damper, full-channel effective, magnetic circuit design, quasi-static model, trapezoidal magnetic ring

## Abstract

Magnetorheological damper (MRD) has been successfully applied to vehicle suspension systems as an intelligent core component. Most conventional MRDs have closed rectangle-shaped magnetic circuits, resulting in a short effective working length and negligible damping force. To address the above issues, a novel full-channel effective MRD with trapezoidal magnetic rings (FEMRD_TMR) is proposed. The trapezoidal magnetic ring can shunt the magnetic circuit, distributing it evenly along the damping channel and increasing the effective working length. Additionally, which has the same variation trend as the magnetic flux through it, makes the magnetic induction intensity distribution more uniform to reduce the magnetic saturation problem. Theoretically analyzing the damping characteristics of the FEMRD_TMR, a quasi-static model is developed to forecast the output damping force. The structural design of MRD is challenging since conventional quasi-static models rely on the yield stress of magnetorheological fluid (MRF) to reflect the rheological property, which cannot be directly observed and is challenging to calculate. The Takagi–Sugeno (T–S) fuzzy neural network and a unique magnetic circuit computation are offered as a novel quasi-static modeling approach to address the issue. The MRF’s yield stress is linearized into magnetic induction intensity functions by the T–S fuzzy neural network and then converted into the MRD’s structural size by the special magnetic circuit calculation. Therefore, the proposed quasi-static model can directly reflect the relationship between the damping force and structure size, simplifying MRD’s structure design. The novel quasi-static model is shown to be more straightforward and understandable than the conventional Bingham quasi-static model and to have approximately accurate damping force prediction when compared to experimental data.

## 1. Introduction

Magnetorheological fluid [1] (MRF) is an intelligent material consisting of magnetizable particles, flow carriers, and stabilizer additives [2]. The magnetorheological damper (MRD), which takes advantage of MRF, has a wide range of adjustable, fast response, low energy consumption, and “fail-safe” characteristics [3]. MRD is an ideal vibration suppression control device that can be used as both passive energy dissipating and control elements [4]. It is widely used in automobiles [5,6], aviation [7], bridges [8], and building [9,10] vibration control.

MRD applied to a vehicle suspension system needs to meet the vehicle’s installation size constraint and have a sufficiently large damping force [11]. Trikand, M.M. [12], applied MRD to an 8 × 8 wheeled armored vehicle and built a 1/4 vehicle testbed. Yoon, D.S. [13] applied MRD to a semi-active suspension system of a passenger car and studied the influence of the MRD’s response time on vibration control. Choi, S.B. [14] designed and fabricated a cylindrical MRD and built an entire vehicle semi-active suspension system with four independent MRDs. Yu, M. [15] developed and manufactured an MRD based on the principle of mixed-mode operation and applied it to a passenger car’s suspension system. All the above scholars have researched the semi-active suspension system based on MRD to improve vehicle ride comfort. However, the traditional MRD still has performance deficiencies, such as insufficient output damping force.

Enhancing the performance of MRD can further improve the suspension system’s ability to attenuate vibration and broaden the engineering application range. Some scholars have researched upgrading MRD’s structure to improve its performance. Jiang, R. [16] developed a novel MRD with selectable performance parameters to improve the environmental adaptability of vibration systems equipped with such a damper. Liu, G. [17] designed and optimized a novel MRD based on a B-spline curve to maximize the controllability of damping force. Lee, T.H. [18] proposed a new magnetic circuit separate from the MRD piston head, which can simplify the coil structure and reduce manufacturing costs. Yuan, X. [19] devised a novel MRD combining a multi-stage circumferential flow mode in external valves. Zhang, J. [20] proposed a novel structure with a trapezoidal cross-section coil for the design of disc-type MR devices to improve the magnetic field distribution. Huang, H. [21] developed a novel MRD to improve the MRF’s settling stability based on the settling principle of the MRF. Yu, J. [22] proposed and analyzed a novel MRD with variable damping and stiffness that contains two driven disks and an active rotary disk. However, most of the MRDs mentioned above require a certain length of excitation coil to generate electromagnetic excitation. Usually, that length will occupy more than 1/2 of the size of the piston. Since the magnetic circuit shape in the MRD piston is an approximate rectangle, no magnetic field lines will pass through the damping channel close to the excitation coil, leading to the problem of short effective working lengths. This problem can lead to low energy efficiency, a small output damping force, and a small adjustable range, limiting MRD’s vibration reduction performance.

Some scholars have researched full-channel effective MRD (FEMRD) to increase the effective working length of MRD. Zheng, L. [23] designed a FEMRD with a stepped non-magnetic ring whose effective working length can reach 90% of the damping channel. Ding, Y. [24] developed a FEMRD with a cone non-magnetic ring, and compared with conventional MRD, the damping force range could increase by more than 70%. Cheng, M. [25,26] proposed a FEMRD with a bent magnetic circuit and performed finite element analysis and tests to verify its effectiveness. The FEMRDs studied above are guided by magnetic and non-magnetic rings for full-channel effectiveness. However, since the magnetic ring is installed in the coil slot, the cross-sectional area is limited, resulting in a small magnetic flux area. A large amount of magnetic flux is required for magnetic rings, which are prone to magnetic saturation problems, especially in small FEMRDs. This problem limits the use of FEMRD in practical engineering applications.

An accurate damping characteristics model is essential when MRD is applied to vehicle suspension systems [27]. The quasi-static model is based on the theory of fluid mechanics, has a rigorous mathematical derivation process, and the model parameters have definite physical significance, usually used in the structural design process of MRD. Wereley, N.M. [28] developed quasi-static MRD models using an idealized Bingham plastic shear flow mechanism, an approximate parallel plate analysis of dashpot dampers was presented, and experiments verified the model’s effectiveness. Dimock, G.A. [29] proposed a Bingham plastic model that can account for both shear thinning and shear thickening behaviors, which assumes a bilinear postyield viscosity with a critical shear rate specifying the region of high shear rate flow. Hong, S.R. [30] utilized the Herschel–Bulkley model to predict fluid flow in a parallel duct with fixed boundaries. Bui, Q.D. [31] combined the quasi-static model of MRD with the magic formula hysteretic multiplier, and the model can be applied to both shear-type and flow-type MRD. Zhang, Z. [32] proposed a physical model of MRD that combines a particle-chain model with a quasi-static model. Research results show that the proposed model accurately describes MRD’s hysteretic properties and maintains better robustness. All the MRD quasi-static models offered by the above scholars rely on the yield stress of MRF to reflect the variation of damping force with magnetic induction intensity. The yield stress of MRF cannot be directly observed and is difficult to calculate, which makes the structural design of MRD difficult.

To solve the above problems, a novel full-channel effective MRD with trapezoidal magnetic rings (FEMRD_TMR) and a novel quasi-static modeling method based on Takagi–Sugeno (T–S) fuzzy neural networks and magnetic circuit calculations are proposed. The FEMRD_TMR uses trapezoidal magnetic rings to shunt the magnetic circuit to achieve full channel efficiency. In addition, the trapezoidal section of the magnetic ring can keep the magnetic flux and the magnetic flux area in the same trend to make the magnetic induction intensity distribution more uniform and reduce the magnetic saturation problem. The effectiveness of the proposed FEMRD_TMR is proven by electromagnetic finite element simulation and experiments. The proposed novel quasi-static modeling method does not rely on the yield stress of MRF. It can directly reflect the relationship between the damping force and the MRD’s structure size, simplifying the MRD’s structure design process. By comparing the experimental data of the FEMRD_TMR, it is proven that the new quasi-static model has higher accuracy than the traditional Bingham quasi-static model.

The paper is organized as follows: Section 2 introduces the basic structure of the FEMRD_TMR. The FEMRD_TMR’s magnetic field lines, magnetic induction intensity, and magnetic field intensity distribution are analyzed by electromagnetic finite element simulation in Section 3. Section 4 introduces the novel quasi-static modeling method. In Section 5, the FEMRD_TMR and the novel quasi-static modeling method are verified and discussed through experiments. Finally, conclusions are shown in Section 6.

## 2. Structure and Principle

**Figure 1:**
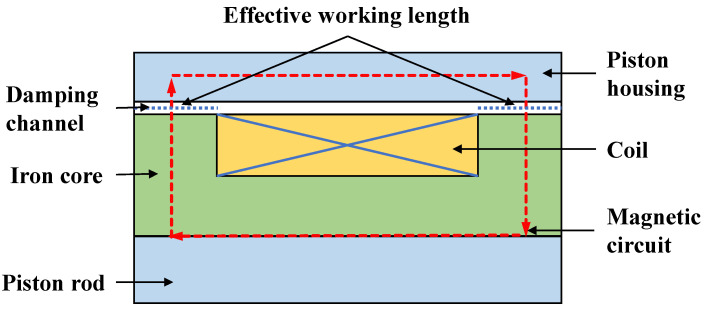
Magnetic circuit diagram of traditional MRD.

The main determinants of MRD’s damping performance are the magnitude and adjustable range of the output damping force. Traditional MRDs [33] require winding the excitation coil in the piston to generate a magnetic field. The length of the excitation coil usually reaches about 1/2 of the total length of the piston. This length range in the damping channel is an invalid working length since no magnetic field lines can flow through it. Therefore, the magnetic circuit shape of conventional MRDs is a simple rectangle with brief working lengths, as shown in Figure 1. Because of this, the output damping force and adjustable range of conventional MRDs are minimal, limiting their ability to reduce vibration.

In order to improve the vibration-damping performance of traditional MRDs, the structure is optimized. The FEMRD_TMR proposed in this paper can shunt the magnetic circuit through the magnetic ring and guide the magnetic field lines through the damping channel within the length range of the excitation coil to achieve full channel efficiency.

**Figure 2:**
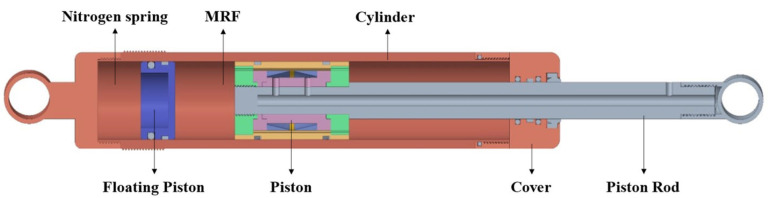
Structure diagram of the FEMRD_TMR.

The structure of the FEMRD_TMR is shown in Figure 2, including the cylinder, nitrogen spring, floating piston, piston, cover, and piston rod. The floating piston slides into the cylinder and separates the nitrogen spring and MRF. The nitrogen spring is filled with nitrogen gas at high pressure to provide compensating force. The piston rod and cylinder are mounted on the frame and wheel. When the vehicle runs, the piston rod and cylinder will produce relative displacement so that the piston moves along the cylinder in the MRF. And then, the FEMRD_TMR generates damping force by squeezing the MRF through the damping channel in the piston.

**Figure 3:**
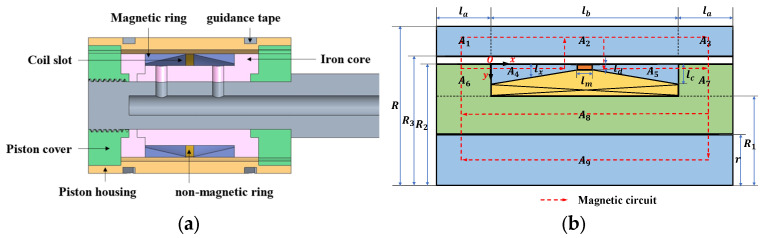
Construction diagram of the piston; (**a**) sectional view; (**b**) schematic diagram.

The piston structure is shown in Figure 3a, including an iron core, guidance tape, magnetic ring, coil slot, piston cover, piston housing, and non-magnetic ring. The dimension marking of the piston section is shown in Figure 3b. The piston is divided into nine areas, where A1, A2, and A3 represent piston housing; A4 and A5 represent trapezoidal magnetic rings; A6, A7, and A8 represent iron core; and A9 stands for the piston rod. $l_{b}$ is the width of the coil slot, $l_{a}$ indicates the length of the partially damped channel in contact with the iron core, and $l_{m}$ and $l_{d}$ display the length and width of the section of the non-magnetic ring. $l_{c}$ represents the width of the bottom edge of the trapezoidal magnetic ring. r, $R_{2}$, $R_{3}$, and R reflects the radius of the piston rod, the radius outside the iron core, the radius inside the piston cover, and the radius inside the cylinder, respectively. $R_{1}$ indicates the radius inside the coil.

Compared with the conventional MRD, the FEMRD_TMR coil slot is equipped with two trapezoidal magnetic rings and a non-magnetic ring. As shown in the magnetic circuit in Figure 3b, the magnetic ring can shunt the magnetic circuit. Part of the magnetic circuit flows from the iron core through the damping channel to the piston housing. The other part flows to the magnetic ring and then through the damping channel to the piston housing, so that all the magnetic field lines pass through the damping channel. In order to avoid magnetic leakage caused by the direct formation of the magnetic circuit between two magnetic rings, a non-magnetic ring is installed. In addition, the magnetic flux through the magnetic ring decreases approximately linearly from the two ends of the damped channel to the middle. The flux area of the trapezoidal magnetic ring can maintain the same linear downward trend, so the magnetic induction intensity can be kept within the saturation value, slowing down the magnetic saturation problem.

## 3. Electromagnetic Finite Element Simulation

An electromagnetic finite element simulation is conducted to analyze the effectiveness of the proposed FEMRD_TMR. The software Maxwell 16.0 used to simulate and analyze the magnetic field lines, magnetic induction intensity, and magnetic field intensity distribution of the FEMRD_TMR. The piston rod, piston cover, and trapezoidal magnetic ring are made of No.45 steel, the iron core material is pure electrician iron DT4, the non-magnetic ring is made of copper, and the coil is coated with copper wire. MRF is MRF-140CG from American Lord Company. The structural dimensions of the FEMRD_TMR, as shown in Table 1. Among them, the cross-sectional area change trend of the trapezoidal magnetic ring needs to be consistent with the declining trend of the passing magnetic flux so that the magnetic induction intensity in the magnetic ring is as close as possible to its saturation magnetic induction intensity of 1.5 T. According to the electromagnetic finite element simulation results and considering the machining difficulty, the size $l_{c}$ and $l_{d}$ of the trapezoidal magnetic ring are selected to be 2.5 mm and 0.2 mm, respectively.

**Figure 4:**
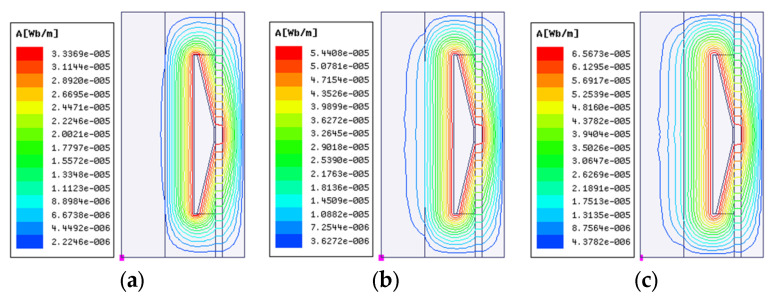
Distribution of magnetic field line under different current excitation. (**a**) 50 A-turns, (**b**) 100 A-turns, (**c**) 150 A-turns.

**Figure 5:**
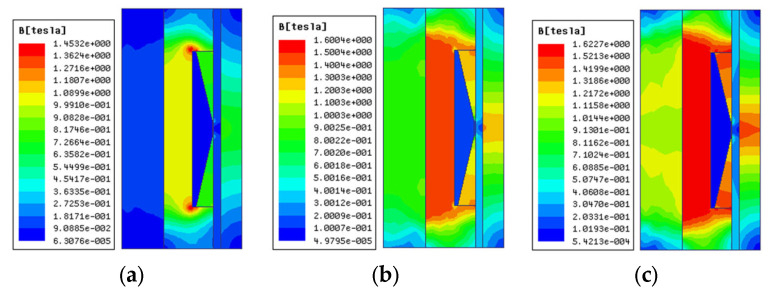
Distribution of magnetic induction intensity under different current excitation. (**a**) 50 A-turns, (**b**) 100 A-turns, (**c**) 150 A-turns.

The coil is a crucial component of MRD. The MRD can obtain the ideal damping performance by regulating the current excitation produced by the coil. First, the maximum current is set at 2 A because high current flow in the coil could pose safety hazards like heating. Then, the distribution of magnetic lines of force and magnetic induction intensity of FEMRD_TMR is investigated using electromagnetic finite element simulation for coil turns of 25, 50, and 75, which means current excitation of 50, 100, and 150 A-turns. The results are displayed in Figure 4 and Figure 5 and Table 2.

The simulation findings show that as the current excitation is increased, the density of the magnetic field line steadily rises and the magnetic field gradually gets stronger. However, the highest magnetic induction intensity of the FEMRD_TMR is 1.6 T, which is just achieved by the saturation magnetic induction intensity of the iron core when the current excitation is 100 A-turns. The maximum magnetic induction intensity of the FEMRD_TMR is 1.62 T, which is higher than the saturation magnetic induction intensity, and this causes a magnetic saturation issue when the current excitation is 150 A-turns. The simulation findings show that 100 A-turns of current can excite the FEMRD_TMR to its maximum potential. Therefore, the coil is most appropriately selected at 50 turns.

**Figure 6:**
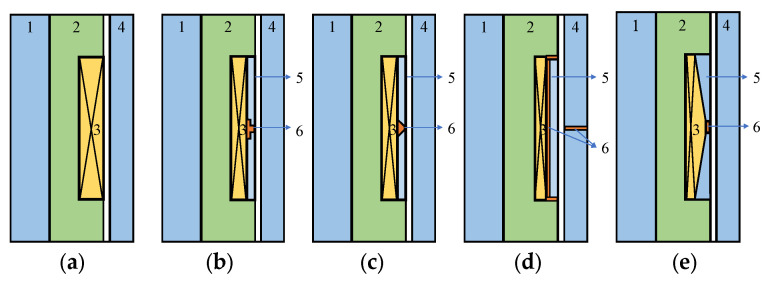
Schematic diagram of five MRDs with different structures. (**a**) type A MRD, (**b**) type B MRD, (**c**) type C MRD, (**d**) type D MRD, (**e**) type E MRD.

Five MRDs are simulated and compared, including ordinary MRD [33], FEMRD with a stepped non-magnetic ring [23], FEMRD with a cone non-magnetic ring [24], FEMRD with a bent magnetic circuit [25], and the proposed FEMRD_TMR, denoted by type A, B, C, D, and E MRD, respectively. The piston structure schematic diagrams are shown in Figure 6. The numbers 1, 2, 3, 4, 5, and 6 indicate the piston rod, iron core, coil, piston housing, magnetic ring, and non-magnetic ring, respectively. Type A MRD has no magnetic conductive structure, and its magnetic circuit will form a closed-loop rectangle around the coil. The structure of type B MRD is similar to that of type C MRD, which has rectangular magnetic structures with a shunt magnetic circuit. Type D MRD achieves full channel effectiveness through magnetic circuit bending. Among the five MRDs, Type A MRD has the easiest coil winding due to its spacious coil slot and simple rectangular coil section. On the other hand, Type E MRD poses the greatest challenge in coil winding as its coil needs to be shaped into a trapezoid and space is limited.

**Figure 7:**
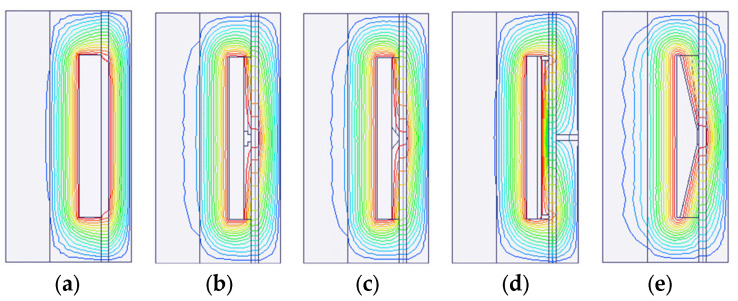
Magnetic field line distribution; (**a**) type A MRD, (**b**) type B MRD, (**c**) type C MRD, (**d**) type D MRD, (**e**) type E MRD.

**Figure 8:**
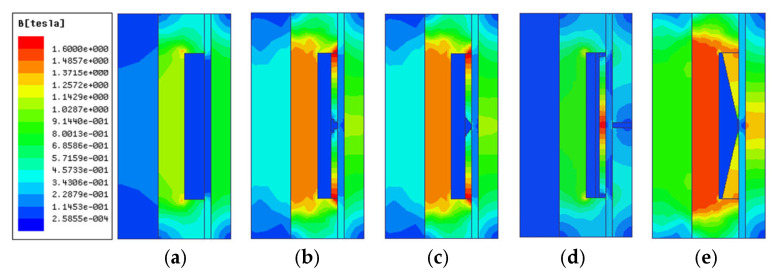
Magnetic induction intensity distribution diagram; (**a**) type A MRD, (**b**) type B MRD, (**c**) type C MRD, (**d**) type D MRD, (**e**) type E MRD.

**Figure 9:**
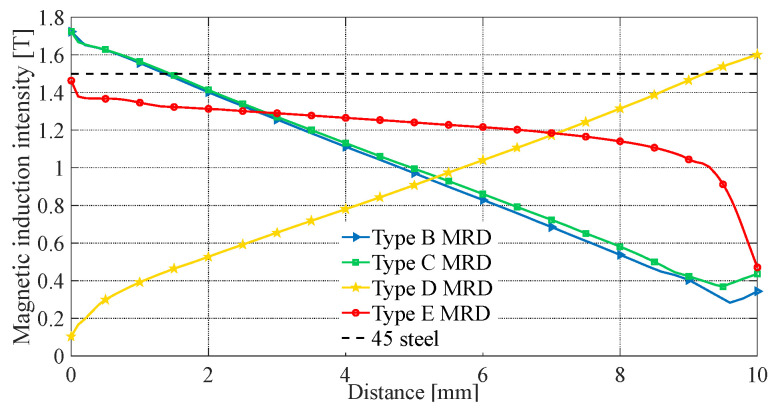
Magnetic induction intensity at magnetic ring.

**Figure 10:**
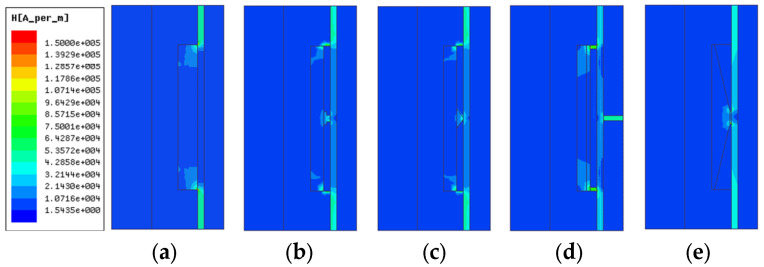
Magnetic field intensity distribution diagram; (**a**) type A MRD, (**b**) type B MRD, (**c**) type C MRD, (**d**) type D MRD, (**e**) type E MRD.

**Figure 11:**
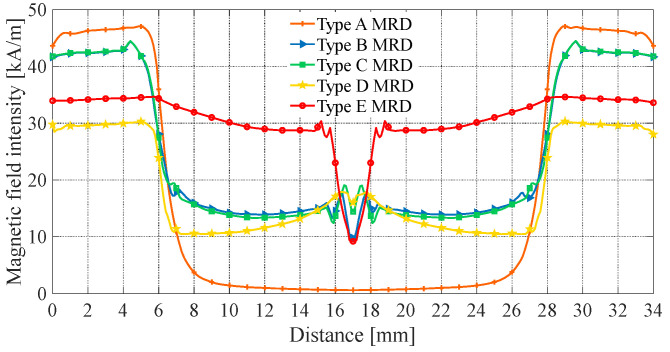
Magnetic field intensity at damping channel.

Under the same structure sizes, material, and current excitation, the electromagnetic finite element simulation results are shown in Figure 7, Figure 8, Figure 9, Figure 10 and Figure 11. The magnetic field line, magnetic induction intensity, and magnetic field intensity distribution of the five MRDs are obtained, respectively.

According to the principle of magnetic circuit design, the magnetic field line should pass through the damping channel as perpendicularly as possible to maximize the magnetic field’s effect on the MRF’s control. As shown in Figure 7, the magnetic field line of all MRDs is perpendicular to the damping channel. However, the magnetic field lines of the type A MRD distribution at the two ends of the damping channel result in a short effective working length. This problem is because the type A MRD needs to wind a coil on the iron core to generate electromagnetic excitation. The damping channel adjacent to the coil has no magnetic field line through it, so it is an invalid working length. Type B, C, D, and E MRD have magnetic field lines distributed throughout the damping channel. Some magnetic field lines of type D MRD directly pass through the non-magnetic ring to form a closed loop without passing through the damping channel, resulting in an apparent magnetic leakage problem. Generally, type B, C, and E MRD’s magnetic field line distribution is more reasonable. The magnetic induction intensity distribution of the five MRDs is shown in Figure 8.

The magnetic induction intensity in all MRD areas must be at most its material’s saturation magnetic induction intensity. Otherwise, the magnetic saturation problem will occur in those areas. It is known that the saturation magnetic induction intensity of the electrical pure iron DT4 is 1.6 T, and No.45 steel is 1.5 T. As seen in Figure 8, the maximum magnetic induction intensity of the type A MRD is 1.31 T at the iron core, and there is no magnetic saturation problem in all areas. The magnetic induction intensity distribution of type B and C MRD is relatively close. The maximum magnetic induction intensity is distributed at the connection between the magnetic ring and iron core, which is 1.73 T, and the magnetic saturation problem exists. The maximum magnetic induction intensity of the type D MRD is 1.61 T at the center of its magnetic ring, which exceeds the saturation magnetic induction intensity of No.45 steel, and the magnetic saturation problem exists. Type E MRD’s maximum magnetic induction intensity is 1.60 T at the iron core, which is equal to the saturation magnetic induction intensity of electrical pure iron DT4. Furthermore, there is no magnetic saturation problem in all areas. So, the magnetic induction intensity distribution of type A and E MRD is reasonable. However, almost all the damping channels of type E MRD have a magnetic induction intensity distribution. In contrast, only the two ends of the damping channel of type A MRD have a magnetic induction intensity distribution. Therefore, the magnetic induction intensity distribution of type E MRD is better.

The magnetic flux area of the magnetic rings in type B, C, and D MRDs remains constant. Moreover, the magnetic flux of type B and C MRD magnetic rings gradually decreases, while that of type D MRD gradually increases, as shown in Figure 9. As a result, the magnetic induction intensity of type B and C MRDs decreases linearly, while that of type D MRD increases linearly. In contrast, the magnetic flux area of the trapezoidal magnetic ring in type E MRD decreases linearly and follows the same trend as the magnetic flux. Therefore, the magnetic induction intensity of type E MRD can more stably remain below the saturation magnetic induction intensity, effectively alleviating the magnetic saturation problem compared to other FEMRDs. This also explains why the maximum magnetic induction of type B, C, and D MRDs occurs in the magnetic ring, while the maximum magnetic induction of type E MRD occurs in the iron core.

The magnetic field intensity distribution of the five MRDs is shown in Figure 10.

The magnetic field intensity at the damping channel directly reflects the magnitude of MRD’s damping force. The effective working length is defined as the part of the damping channel in which the magnetic field intensity is greater than 10 kA/m. It can be seen from Figure 9 and Figure 11 that the magnetic field intensity of the type A MRD’s damping channel is distributed at both ends. Calculate the effective working length of the type A MRD, which only accounts for 41.18% of the damping channel. The maximum magnetic field intensity of type B and C MRD is between 41 and 44 kA/m on both ends of the damping channel, and the damping channel near the coil is between 13 and 20 kA/m. The magnetic field intensity at both ends of the type D MRD’s damping channel is between 28 and 30 kA/m, and the damping channel near the coil is between 10 and 18 kA/m, which is caused by the fact that the type D MRD has an evident magnetic leakage problem. The magnetic field intensity distribution of the type E MRD’s damping channel is the most uniform, ranging from 28 to 37 kA/m, and the effective working length accounts for 99.12%. The average magnetic field intensity of the five MRDs’ damping channels is calculated as shown in Table 3.

For the type E MRD, the magnetic flux area is larger at the connection between the trapezoidal magnetic ring and the iron core, which can attract more magnetic flux through the trapezoidal magnetic ring for the magnetic shunt. So, increasing the magnetic flux of the damping channel near the coil makes the magnetic field intensity more uniform and improves the average magnetic field intensity. As shown in Table 3, the average magnetic field intensity of the type E MRD’s damping channel is the largest, at 30.65 kA/m, and the type A MRD is the smallest, at only 18.52 kA/m. The output damping force of MRD is positively correlated with the magnetic field intensity at the damping channel. So, under the same current excitation, the damping channel of type E MRD has a more significant average magnetic field intensity, a larger output damping force, and a higher energy utilization rate than other MRDs.

## 4. Quasi-Static Model

The quasi-static model is typically used in the structural design phase of MRD. It is based on the theory of fluid mechanics, has a rigorous mathematical derivation method, and the model parameters have clear physical significance and have the advantages of simplicity and low computational cost. Traditional quasi-static models rely on the yield stress of MRF to reflect the variation of damping force with magnetic induction intensity. The yield stress of MRF cannot be directly observed and is difficult to calculate, which makes the structural design of MRD difficult.

This paper proposes a novel quasi-static modeling method to simplify the structural design of MRD. The yield stress of MRF is converted into the structural dimension parameters of MRD by using a T–S fuzzy neural network and a special magnetic circuit calculation. The corresponding relationship between damping force and structural dimension is established to simplify the structural design process of MRD.

For flow-mode MRD, its damping force F can be decomposed into the magnetorheological viscous damping force $F_{\tau }$ controlled by yield stress τ, and the Newtonian damping force $F_{\mu }$ caused by liquid viscosity and friction [3], as shown in Equation (1):

(1) $$F=F_{\mu }+F_{\tau }.$$

MRD adjusts the damping characteristics by changing the solid–liquid state of MRF, so the flow state of MRF must be clear. It is assumed that the piston speed of the FEMRD_TMR is constant and that MRF flows entirely. According to hydrodynamics, fluid flow in the annular gap can be treated as a parallel plate flow model under quasi-static conditions [28]. The viscous pressure drop $\Delta p_{\mu }$ is represented as:

(2) $$\Delta p_{\mu }=\frac{6\mu lQ}{\pi R_{2}h^{3}},$$

where μ is the viscosity coefficient of MRF, Q is the flow through the damping channel, l is the effective working length of the damping channel, and h is the damping channel width.

The FEMRD_TMR is able to achieve fully damped channels with effective working lengths, the l can be calculated by:

(3) $$l=2l_{a}+l_{b}.$$

And the calculation of Q is simplified as:

(4) Q=ΔA⋅v,

where ΔA represents the effective working section area of the piston, and its calculation formula is:

(5) $$\Delta A=\pi \left(R^{2}-\left(R_{3}{}^{2}-R_{2}{}^{2}\right)-r^{2}\right).$$

By combining Equations (1)–(5), the formula for calculating the Newtonian damping force $F_{\mu }$ is given by:

(6) $$F_{\mu }=\Delta A\cdot \Delta p_{\mu }==\frac{6\pi \mu \left(2l_{a}+l_{b}\right){\left(R^{2}-\left(R_{3}{}^{2}-R_{2}{}^{2}\right)-r^{2}\right)}^{2}v}{R_{2}h^{3}}.$$

According to the reference [34], the pressure drops due to the yield stress $\Delta p_{\tau }$ is given by:

(7) $$\Delta p_{\tau }=\frac{A_{1}}{A_{2}}\tau_{0}\cdot \mathrm{sgn}(v),$$

where, $\tau_{0}$ is the yield stress of MRF, $A_{1}$ is the equivalent area of the friction surface of the damping channel, and $A_{2}$ is the cross-sectional area of the damping channel, they are calculated by:

(8) $$A_{1}=\pi \left(R_{3}+R_{2}\right)cl,$$

(9) $$A_{2}=\pi \left(R_{3}{}^{2}-R_{2}{}^{2}\right)=\pi \left(R_{3}+R_{2}\right)\left(R_{3}-R_{2}\right)=\pi \left(R_{3}+R_{2}\right)h,$$

where, c is defined as the magnetorheological effect coefficient, which has a value ranging from a minimum value of 1 (for $\Delta p_{\tau }/\Delta p_{\mu }<1$) to a maximum value of 3 (for $\Delta p_{\tau }/\Delta p_{\mu }>100$) [35]. It follows that:

(10) $$\Delta p_{\tau }=\frac{c\left(2l_{a}+l_{b}\right)\tau_{0}}{h}\cdot \mathrm{sgn}(v).$$

The formula for calculating the magnetorheological viscous damping force $F_{\tau }$ is given by:

(11) $$F_{\tau }=\Delta A\cdot \Delta p_{\tau }=\pi \left(R^{2}-\left(R_{3}{}^{2}-R_{2}{}^{2}\right)-r^{2}\right)\frac{c\left(2l_{a}+l_{b}\right)\tau_{0}}{h}\cdot \mathrm{sgn}(v).$$

Combined with Equations (1)–(11), the theoretical damping force of the FEMRD_TMR can be calculated as follows:

(12) $$F=\pi \left(R^{2}-\left(R_{3}{}^{2}-R_{2}{}^{2}\right)-r^{2}\right)\left(2l_{a}+l_{b}\right)\left(\frac{6\mu \left(R^{2}-\left(R_{3}{}^{2}-R_{2}{}^{2}\right)-r^{2}\right)v}{R_{2}h^{3}}+\frac{c\tau_{0}}{h}\mathrm{sgn}(v)\right).$$

In Equation (12), it is not easy to calculate the variable $\tau_{0}$, which depends on the MRF’s material properties and magnetic induction intensity. If the variable $\tau_{0}$ can be eliminated, then the variable in the formula will only be the MRD’s structural size at a given velocity. So, it can intuitively calculate the damping force change when the MRD’s structure size changes. In order to achieve this goal, the quasi-static modeling method proposed in this paper uses a T–S fuzzy neural network to linearize the relationship between $\tau_{0}$ and magnetic induction intensity and then combines it with the theoretical calculation of the shunted magnetic circuit to obtain the magnetic induction intensity of the damping channel, thus converting the variable $\tau_{0}$ into the MRD’s structure size.

**Figure 12:**
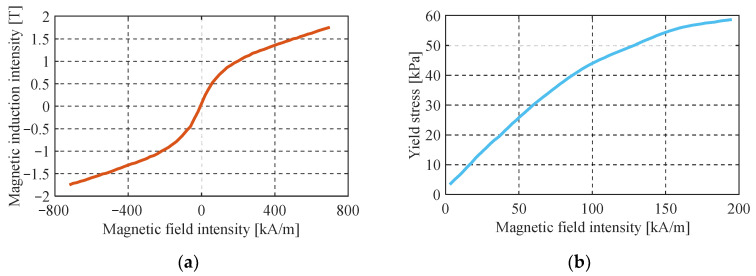
Rheological properties curve of MRF; (**a**) magnetic induction intensity versus magnetic field intensity; (**b**) yield stress versus magnetic field intensity.

**Figure 13:**
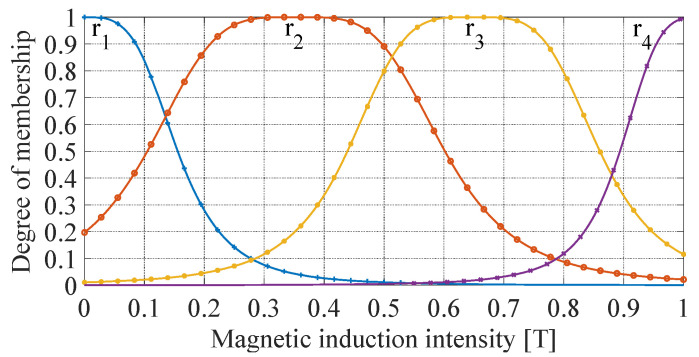
Generalized bell-shaped affiliation function of the T–S fuzzy neural network.

Take MRF-140CG magnetorheological fluid produced by the Lord Company of the United States as an example. Its rheological properties are shown in Figure 12:

The T–S fuzzy neural network is used to express the relationship between yield stress and magnetic induction intensity. As shown in Figure 13, select the generalized bell-shaped affiliation function and take four fuzzy subsets to cover the magnetic induction intensity.

The expression of the generalized bell-shaped affiliation function $r_{i}\left(i=1,2,3,4\right)$ is:

(13) $$r_{i}=\frac{1}{1+{\left|\frac{B_{f}-c_{i}}{a_{i}}\right|}^{2b_{i}}},$$

where $B_{f}$ is the magnetic induction intensity, $a_{i}\;$, $b_{i}$ and $c_{i}$ are constants.

The fuzzy rule obtained is

(14) $$\mathrm{Fuzzy Rule}\;i:\;\mathrm{IF}\;B_{f}\;\mathrm{is}\;r_{i},\;\;\mathrm{THEN}\;u_{i}=d_{i}B_{f}+e_{i}.$$

where $u_{i}\left(i=1,2,3,4\right)$ is the output result corresponding to the above fuzzy rules, $d_{i}$ and $e_{i}$ are constants. $a_{i},\;b_{i},c_{i},\;d_{i}$ and $e_{i}$ their values are shown in Table 4.

The yield stress $\tau_{0}$ can be obtained by the weighted average method:

(15) $$\tau_{0}=\sum_{i=1}^{4}q_{i}r_{i}u_{i},$$

where, $q_{i}$ is the weight factor, which is a constant used to adjust the weight of each rule in the T–S fuzzy neural network.

In Equation (15), the yield stress $\tau_{0}$ has been transformed into a linear function of magnetic induction intensity $B_{f}$. Then $B_{f}$ can be obtained by a theoretical calculation of the shunted magnetic circuit.

The magnetic induction intensity of the FEMRD_TMR is calculated according to the ampere–loop theorem and the Gauss theorem. As shown in Figure 3b, the piston is divided into nine regions: A1~A9. Calculate the magnetic flux in each area as follows:

(16) $$\phi_{1}=\phi_{3}=B_{1}S_{1}=B_{1}\cdot \pi \left(R+R_{3}\right)\sqrt{{\left(R-R_{3}\right)}^{2}+l_{a}{}^{2}},$$

(17) $$\phi_{2}=B_{2}S_{2}=B_{2}\cdot \pi \left(R^{2}-R_{3}{}^{2}\right),$$

(18) $$\phi_{6}=\phi_{7}=B_{6}S_{6}=B_{6}\cdot \pi \left(R_{1}+R_{2}\right)l_{a},$$

(19) $$\phi_{8}=B_{8}S_{8}=B_{8}\cdot \pi \left(R_{1}{}^{2}-r^{2}\right),$$

(20) $$\phi_{9}=B_{9}S_{9}=B_{8}\cdot \pi r^{2},$$

(21) $$\phi_{f}=B_{f}S_{f}=B_{f}\cdot \pi \left(\frac{R_{2}+R_{3}}{2}\right)\left(2l_{a}+l_{b}\right),$$

where $\phi_{i}(i=1,2,\ldots ,9)$ are the magnetic flux passing through each region, $B_{i}$ are the corresponding magnetic induction intensity, $S_{i}$ are the corresponding magnetic flux area, $\phi_{f}$ is the magnetic flux through the damping channel, and $S_{f}$ is the magnetic flux area of the damping channel.

The magnetic flux through the trapezoidal magnetic ring is calculated. As shown in Figure 3b, when the coordinate system is set at the origin of point O where the magnetic ring connects with the iron core, x and y are respectively set as the length and width of the trapezoidal magnetic ring, have:

(22) $$\phi_{4x}=\phi_{5x}=B_{4x}S_{4x}=B_{4x}\cdot \pi \left(R_{2}{}^{2}-{\left(R_{2}-l_{x}\right)}^{2}\right),$$

where $\phi_{4x}$ and $\phi_{5x}$ represents the magnetic flux through the trapezoidal magnetic ring at the distance x from the origin, $B_{4x}$ represents the corresponding magnetic induction intensity, $S_{4x}$ represents the corresponding magnetic flux area. Then, the maximum magnetic flux $\phi_{4max}$, $\phi_{5max}$ and the minimum magnetic flux $\phi_{4min}$, $\phi_{5min}$ of the trapezoidal magnetic ring are:

(23) $$\phi_{4max}=\phi_{5max}=B_{4c}\cdot \pi \left(R_{2}{}^{2}-{\left(R_{2}-l_{c}\right)}^{2}\right),$$

(24) $$\phi_{4min}=\phi_{5min}=B_{4d}\cdot \pi \left(R_{2}{}^{2}-{\left(R_{2}-l_{d}\right)}^{2}\right),$$

where $B_{4c}$ represents the magnetic induction intensity at the connection between the magnetic ring and the iron core; $B_{4d}$ represents the magnetic induction intensity at the connection between the magnetic ring and the non-magnetic ring.

According to the conservation of magnetic flux according to the Gauss theorem, it can be obtained that:

(25) $$\phi_{f}=\phi_{6}=\phi_{7}=\phi_{2}=\phi_{1}+\phi_{4max}=\phi_{3}+\phi_{5max}=\phi_{8}+\phi_{9}.$$

According to Equation (25), the magnetic flux passing through A2, A6, and A7 is the largest, and the magnetic flux area in A2 is the smallest, so region A2 is the first to reach magnetic saturation. In the structural design process, the magnetic induction intensity of A2 can be assumed to be the saturation magnetic induction intensity $B_{2\max}$, and thus $\phi_{2}$ can be obtained, and $\phi_{f}$ can be calculated:

(26) $$\phi_{f}=\phi_{2}=B_{2\max}\cdot \pi \left(R^{2}-R_{3}{}^{2}\right).$$

Then, $B_{f}$ can be calculated by:

(27) $$B_{f}=\frac{\phi_{2}}{\pi \left(\frac{R_{2}+R_{3}}{2}\right)\left(2l_{a}+l_{b}\right)}.$$

The material of A2 is No.45 steel, whose maximum magnetic induction intensity $B_{2\max}$ is 1.5 T. $B_{f}$ is 0.304 T according to the structural size of the FEMRD_TMR in Table 1 and calculated by Equation (27). According to Figure 8, the simulation result of the average magnetic induction intensity of the FEMRD_TMR damping channel is 0.312 T. It can be seen that the magnetic induction intensity obtained by the theoretical calculation method of the magnetic circuit has a minor error with the simulation result; the magnetic circuit calculation method has a high calculation accuracy.

So that the quasi-static model of the FEMRD_TMR can be written as follows:

(28) $$\left\{\begin{array}{c} F=\pi \left(R^{2}-\left(R_{3}{}^{2}-R_{2}{}^{2}\right)-r^{2}\right)\left(2l_{a}+l_{b}\right)\left(\frac{6\mu \left(R^{2}-\left(R_{3}{}^{2}-R_{2}{}^{2}\right)-r^{2}\right)v}{R_{2}h^{3}}+\frac{c(\sum_{i=1}^{4}q_{i}r_{i}u_{i})\mathrm{sgn}(v)}{h}\right)\\ r_{i}=\frac{1}{1+{\left|\frac{B_{f}-c_{i}}{a_{i}}\right|}^{2b_{i}}}\\ u_{i}=d_{i}B_{f}+e_{i}\\ B_{f}=\frac{B_{2\max}\left(R^{2}-R_{3}{}^{2}\right)}{\frac{R_{2}+R_{3}}{2}\left(2l_{a}+l_{b}\right)} \end{array}\right.$$

As can be seen from Equation (28), under certain operating conditions, the velocity v is a constant value, and the variable in the calculation formula for the theoretical damping force F will only be the MRD’s structure sizes. In the structural design of MRD, the quasi-static model can be used to intuitively calculate the damping force change when the MRD’s structure size changes to simplify the MRD’s structure design process. The quasi-static modeling method based on T–S fuzzy neural networks and magnetic circuit calculations can also be applied to the traditional flow-type MRD.

## 5. Experimental Verification

### 5.1. Prototype and Test System Setup

**Figure 14:**
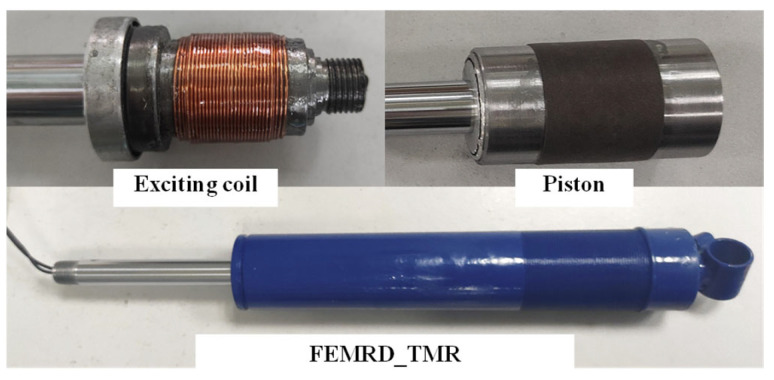
Machining and assembly drawings.

The FEMRD_TMR is processed and assembled, as shown in Figure 14. To further verify its effectiveness, the damping characteristic test of the FEMRD_TMR is carried out.

**Figure 15:**
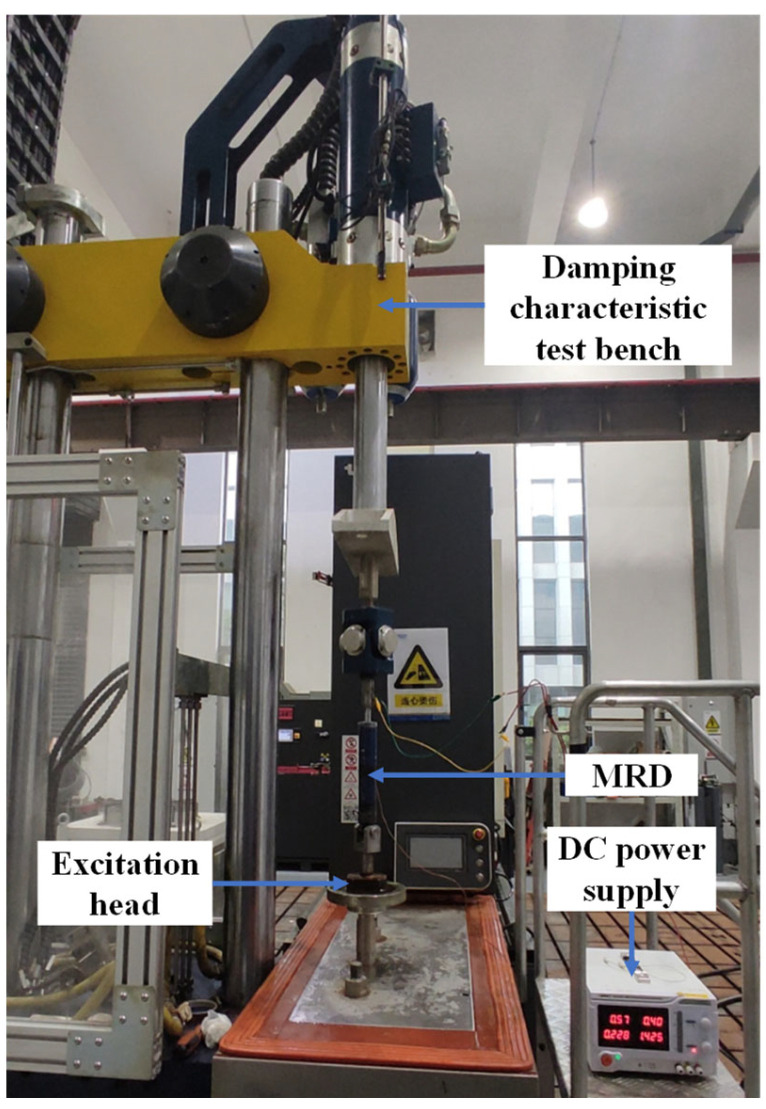
Damping characteristics test.

As shown in Figure 15, the test equipment includes the SVT-DTS-50 damping characteristic test bench and DC power supply. The damping characteristic test bench is integrated with excitation, sensing, and signal acquisition systems. The DC power supply is used to supply current to the FEMRD_TMR. A computer is used to adjust the input signal of the excitation head and observe the test results.

### 5.2. Damping Characteristic Analysis

Sinusoidal excitation is used in the testing of damping characteristics. Based on the typical operating conditions of the vehicle suspension system, the test conditions are selected as follows:

- The peak velocity of the sinusoidal excitation is 0.1 m/s, 0.3 m/s, and 0.6 m/s, respectively.
- The test stroke is ±15 mm.
- The excitation current is 0 A, 0.4 A, 0.8 A, 1.2 A, 1.6 A, 2.0 A, and 3.0 A, respectively.

**Figure 16:**
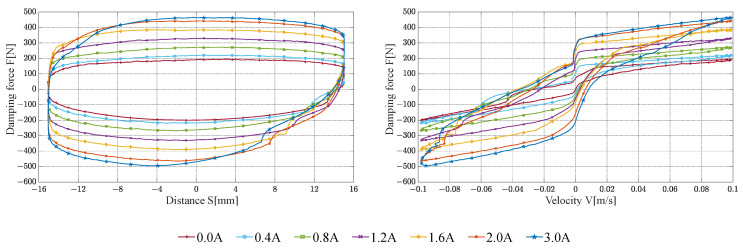
Damping characteristic at a peak velocity of 0.1 m/s.

**Figure 17:**
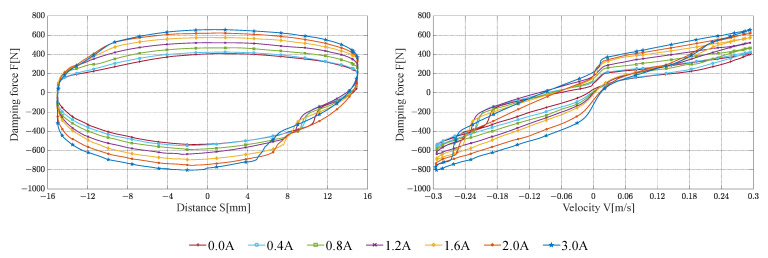
Damping characteristic at a peak velocity of 0.3 m/s.

**Figure 18:**
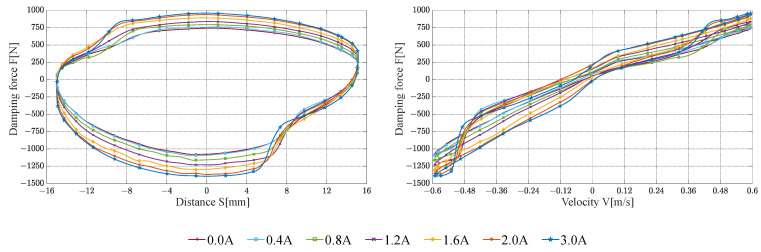
Damping characteristic at a peak velocity of 0.6 m/s.

The damping characteristic test results of the FEMRD_TMR are shown in Figure 16, Figure 17 and Figure 18.

When the input current is 2.0 and 3.0 A, the maximum damping force of the FEMRD_TMR comes close to 1370 N and 1395 N, respectively. That is because the FEMRD_TMR has reached magnetic saturation within this current range. When the current is 0.0 A and the peak velocity is 0.1 m/s, the maximum damping force is 200 N. When the current is 3.0 A and the peak velocity is 0.6 m/s, the maximum damping force is 1395 N. The FEMRD_TMR has a wide range of damping forces. In Figure 18, there is a sag phenomenon on both sides of the relationship curve between damping force and displacement, which becomes more obvious with the increase in peak excitation velocity. The hysteresis of the floating piston causes this phenomenon, and the static friction between the floating piston and cylinder is changed into dynamic friction, resulting in a sudden change of damping force.

The FEMRD_TMR is compared with the ordinary MRD in reference [33], as shown in Table 5.

The maximum damping force of the ordinary MRD with a cylinder diameter of 62 mm is 1378 N when the power excitation is 150 A-turns. The proposed FEMRD_TMR has a cylinder diameter of 42 mm, and its maximum damping force is 1395 N under the same working conditions. Therefore, compared with the ordinary MRD and the FEMRD with a bent magnetic circuit, the proposed FEMRD_TMR can have a more significant damping force with a smaller structure size.

### 5.3. Quasi-Static Model Validation

**Figure 19:**
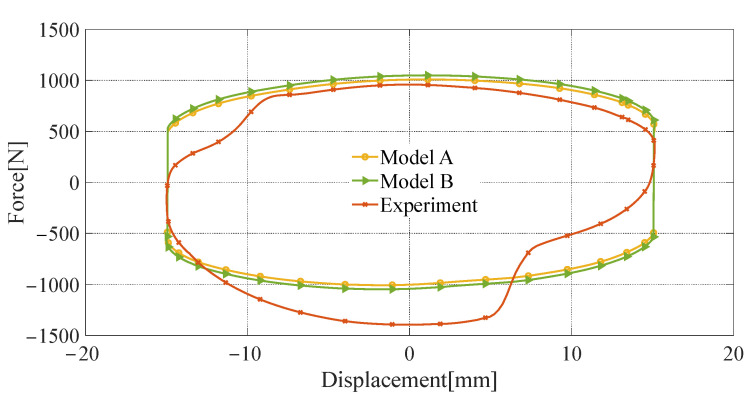
Comparison of quasi-static model and experimental results (current 3 A, peak velocity 0.6 m/s).

**Figure 20:**
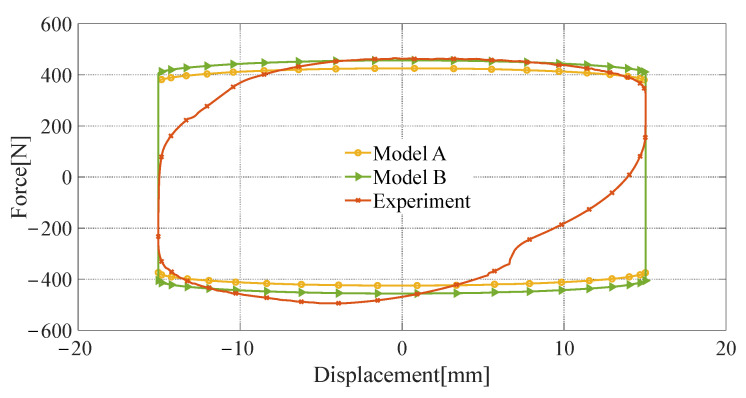
Comparison of quasi-static model and experimental results (current 3 A, peak velocity 0.1 m/s).

**Figure 21:**
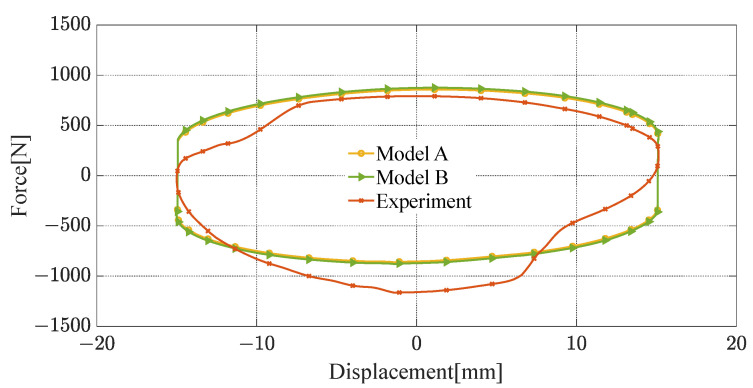
Comparison of quasi-static model and experimental results (current 0.8 A, peak velocity 0.6 m/s).

The novel quasi-static model proposed in this paper (referred to as model A) is validated by comparing it with the Bingham quasi-static model [34] (referred to as model B), using the test results from the aforementioned damping characteristics experiments. The Bingham quasi-static model is a widely used quasi-static modeling method for representing the rheological properties of MRF through the Bingham operator, which is expressed in Equation (29). However, in model B, the yield stress of the MRF cannot be directly obtained, and reference to the magnetic characteristic curve as depicted in Figure 12 becomes necessary. The comparison results are presented in Figure 19, Figure 20 and Figure 21.

(29) $$F=\frac{32\mu (2l_{a}+l_{b})}{h^{2}}(\frac{\Delta A^{2}}{\pi R_{2}h})v+\frac{16\tau_{0}(2l_{a}+l_{b})}{h}\Delta A\cdot \mathrm{sgn}(v)$$

The root-mean-square error (RMSE) of model A and experimental results are denoted as RMSE A, and RMSE B is the RMSE of model B and experimental results. As shown in Table 6, when the input current is 3 A and the peak velocity of sinusoidal excitation is 0.1 m/s, RMSE A is 176.84 N, and RMES B is 192.53 N, model A has a lower RMSE than model B by 8.15%. When the input current is 3 A and the peak speed is 0.6 m/s, the RMSE for models A and B are 281.57 N and 298.61 N, respectively, and model A has a 5.71% reduction in RMSE relative to model B. When the input current is 0.8 A and the peak velocity is 0.6 m/s, RMSE A and RMES B are 218.20 N and 226.65 N and model A has a lower RMSE than model B by 3.73%. RMSE A and RMSE B decrease with the input current, or peak velocity.

The modeling accuracy of model A is slightly higher than model B’s because the magnetic circuit calculation method with shunt characteristics adopted in model A is more consistent with the actual magnetic circuit characteristics of the FEMRD_TMR.

## 6. Conclusions

This research proposes a novel full-channel effective magnetorheological damper with trapezoidal magnetic rings. The trapezoidal magnetic ring is used to divide the magnetic circuit and increase the effective working length of the damping channel. Then, a quasi-static modeling method based on a T–S fuzzy neural network and a special magnetic circuit calculation are proposed to simplify the structural design process of the FEMRD_TMR. The investigated results are summarized as follows:

1. The proposed FEMRD_TMR can increase the effective working length of the damping channel up to 99.12%, reduce magnetic saturation problems, and improve the average magnetic field intensity.
2. Compared with type A, B, C, and D MRD, the FEMRD_TMR can output a larger damping force with a smaller structure size, which is more in line with the actual engineering requirements of the vehicle suspension system and widens the application range of MRD in miniature vehicles.
3. The proposed quasi-static modeling method does not rely on the yield stress of MRF. It can directly reflect the relationship between the damping force and MRD’s structure size, which makes the structural design of MRD simpler. And the novel quasi-static modeling method has higher accuracy than the traditional Bingham quasi-static modeling method.

## Figures and Tables

**Table 1:** Structural parameters of the FEMRD_TMR.

**Parameters**	$l_{a}/$ **mm**	$l_{b}/$ **mm**	r/ **mm**	$R_{1}/$ **mm**	$R_{2}/$ **mm**
Values	6	22	6	10	13
**Parameters**	$R_{3}/$ **mm**	R/ **mm**	$l_{c}/$ **mm**	$l_{d}/$ **mm**	$l_{m}/$ **mm**
Values	14	17	2.5	0.2	2

**Table 2:** Magnetic field line density and magnetic induction intensity under different current excitation.

Current Excitation (A-Turns)	50	100	150
Magnetic field line density (Wb/m)	3.34	5.44	6.57
Magnetic induction intensity (T)	1.45	1.60	1.62

**Table 3:** Average magnetic field intensity at damping channel.

MRD Type	Type A	Type B	Type C	Type D	Type E
Average magnetic field intensity (kA/m)	18.52	24.62	24.50	18.69	30.65

**Table 4:** T–S fuzzy neural network parameters.

Rulei	1	2	3	4
Parameter $a_{i}$	0.17	0.25	0.21	0.14
Parameter $\;b_{i}$	2.00	2.00	2.00	2.00
Parameter $\;c_{i}$	0.00	0.35	0.65	1.04
Parameter $d_{i}$	−11.65	34.84	44.47	18.85
Parameter $e_{i}$	1.74	1.66	14.37	40.20

**Table 5:** MRD comparison of different structures.

MRD Type	Ordinary MRD	FEMRD_TMR
Cylinder diameter(mm)	62	42
Power excitation (A-turns)	150	150
Maximum damping force(N)	1378	1395

**Table 6:** Root mean square error of quasi-static models and experimental.

Current (A)	Peak Velocity (m/s)	RMSE A (N)	RMSE B (N)
3	0.1	176.84	192.53
3	0.6	281.57	298.61
0.8	0.6	218.20	226.65

## Data Availability

Data sharing is not applicable for this article.
